# Supplementary material for: Arbuscular Mycorrhiza Support Plant Sulfur Supply through Organosulfur Mobilizing Bacteria in the Hypho- and Rhizosphere
Source: Plants (Basel). 2022 Nov 11;11(22):3050. doi: 10.3390/plants11223050 (PMC9694294; doi:10.3390/plants11223050)
Supplement: Supplementary file 1 [file plants-11-03050-s001.zip › plants-2027182-supplementary.pdf]

Arbuscular Mycorrhiza Support Plant Sulfur Supply through Organosulfur Mobilizing Bacteria in the Hypho- and Rhizosphere

Jacinta Gahan, Orla O'Sullivan, Paul D. Cotter and Achim Schmalenberger

Supplementary Material

Supplementary figures S1-S8

Supplementary information S1-S5

Supplementary table S1

Supplementary figures:

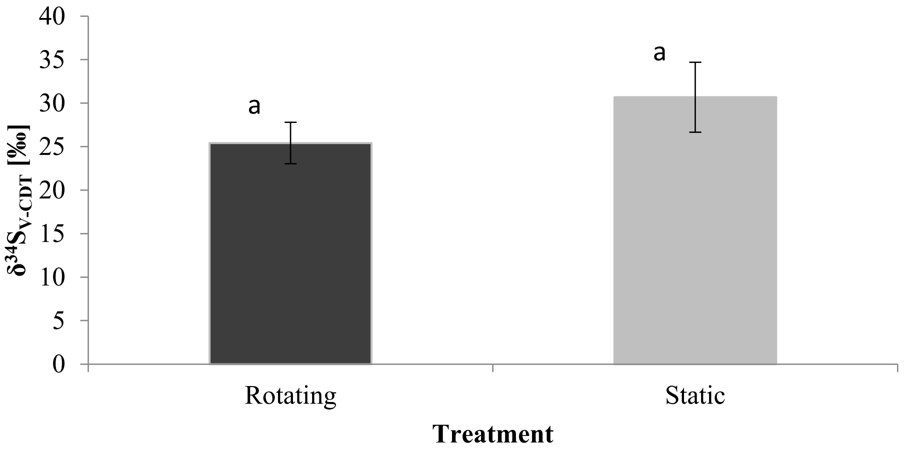

Figure S1 – AM uptake of ^{34}S from organo-^{34}S after 12 months (3 months) post transfer into new *Agrostis stolonifera* microcosms. Rotating = severed hyphae, Static = mycorrhizal. No significant differences (a).

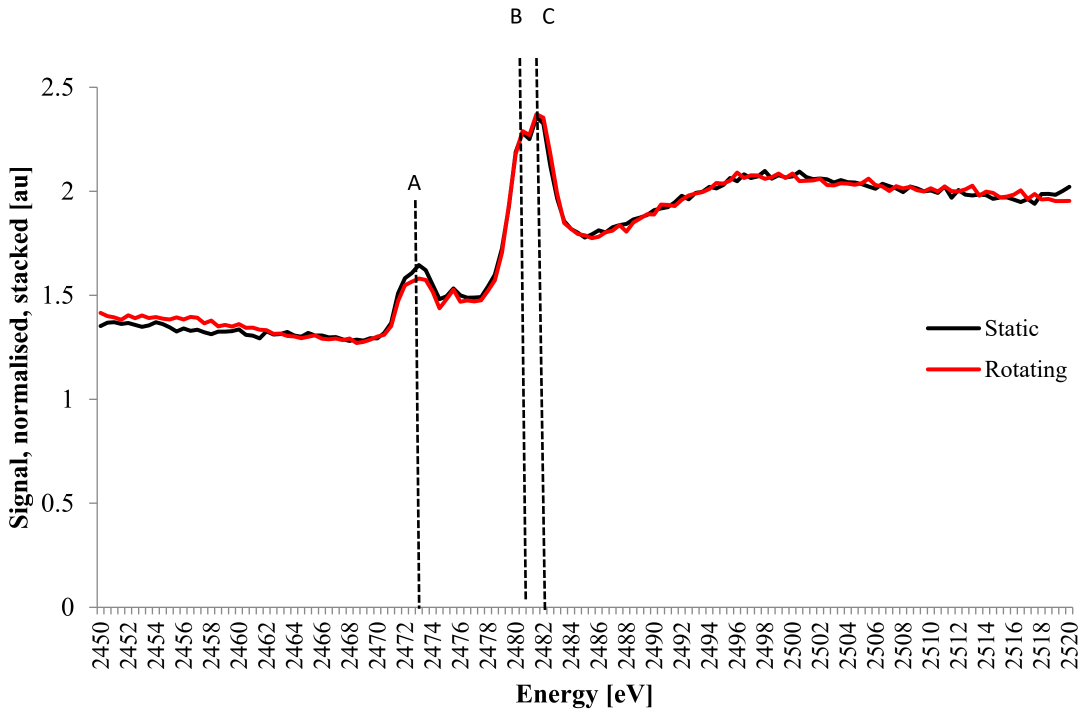

Figure S2 – Normalised and stacked S K-edge spectra from static (black) and rotating (red) treatments with *Agrostis stolonifera* as host plant. Peak A represents reduced thiols, peak B represents intermediate sulfonates and peak C represents oxidised sulfate esters [30].

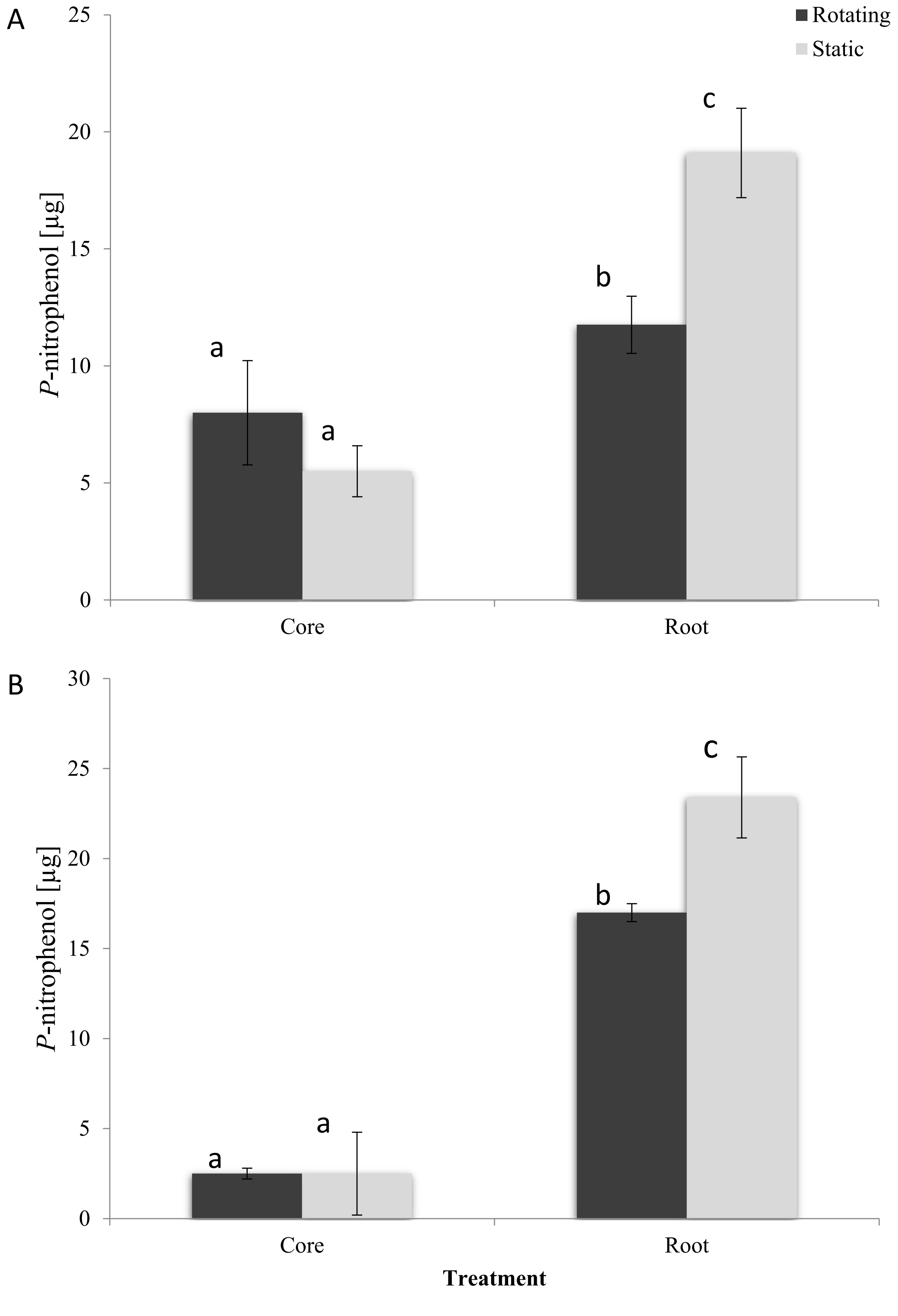

Figure S3 – Arylsulfatase activity for *Agrostis stolonifera* (A) and *Plantago lanceolata* (B) in organo-^{34}S enriched soil microcosm systems. Rotating = severed hyphae, static = mycorrhizal, roots = rhizosphere, and cores = hyphosphere. Letters (a-c) represent significant differences.

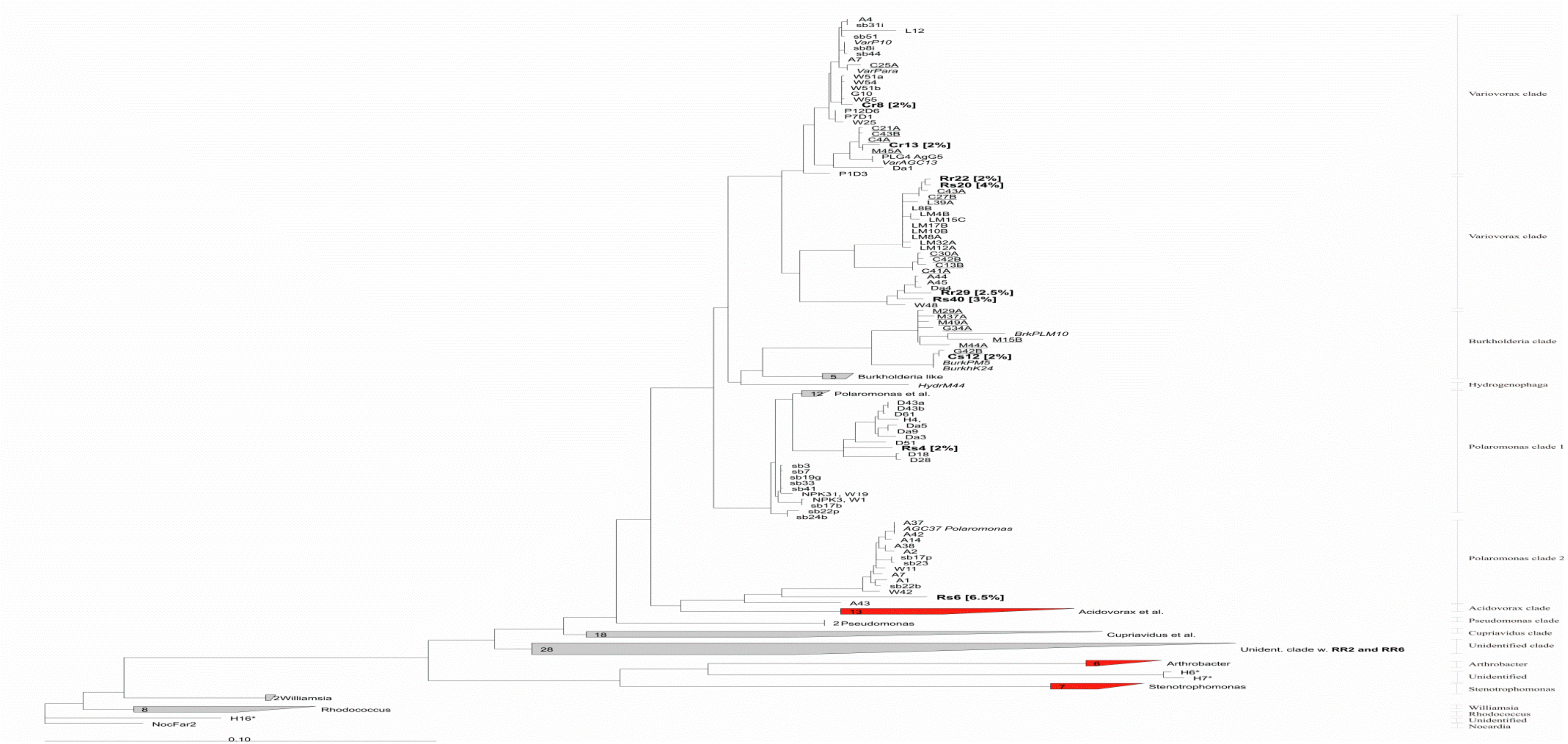

Figure S4 – Randomised axelerated maximum likelihood tree of truncated AsfA sequences of representative desulfonating operational taxonomic units (OTUs) derived from clonal *asfA* DNA sequence analysis from static (CS) and rotating (CR) hyphosphere cores and rhizosphere roots from static = mycorrhizal (RS) and rotating = severed mycorrhizal hyphae (RR) treatments of *A. stolonifera* microcosms. Cultivated (*italics*) and molecular isolates from this study (bold) are highlighted. Molecular isolates from spring barley rhizospheres (sb; [11]), *Agrostis* grassland rhizospheres (CA; [43), wheat rhizospheres from Broadbalk (W; [12]}), rhizospheres and soils from the Damma glacier forefield (D; DA; [61]) and hyphosphere (H; [15]) were isolated previously. Clades expanded include *Variovorax, Burkholderia,* and *Polaromonas.* Clades to be expanded in the figure below (Figure S5) are highlighted in red.

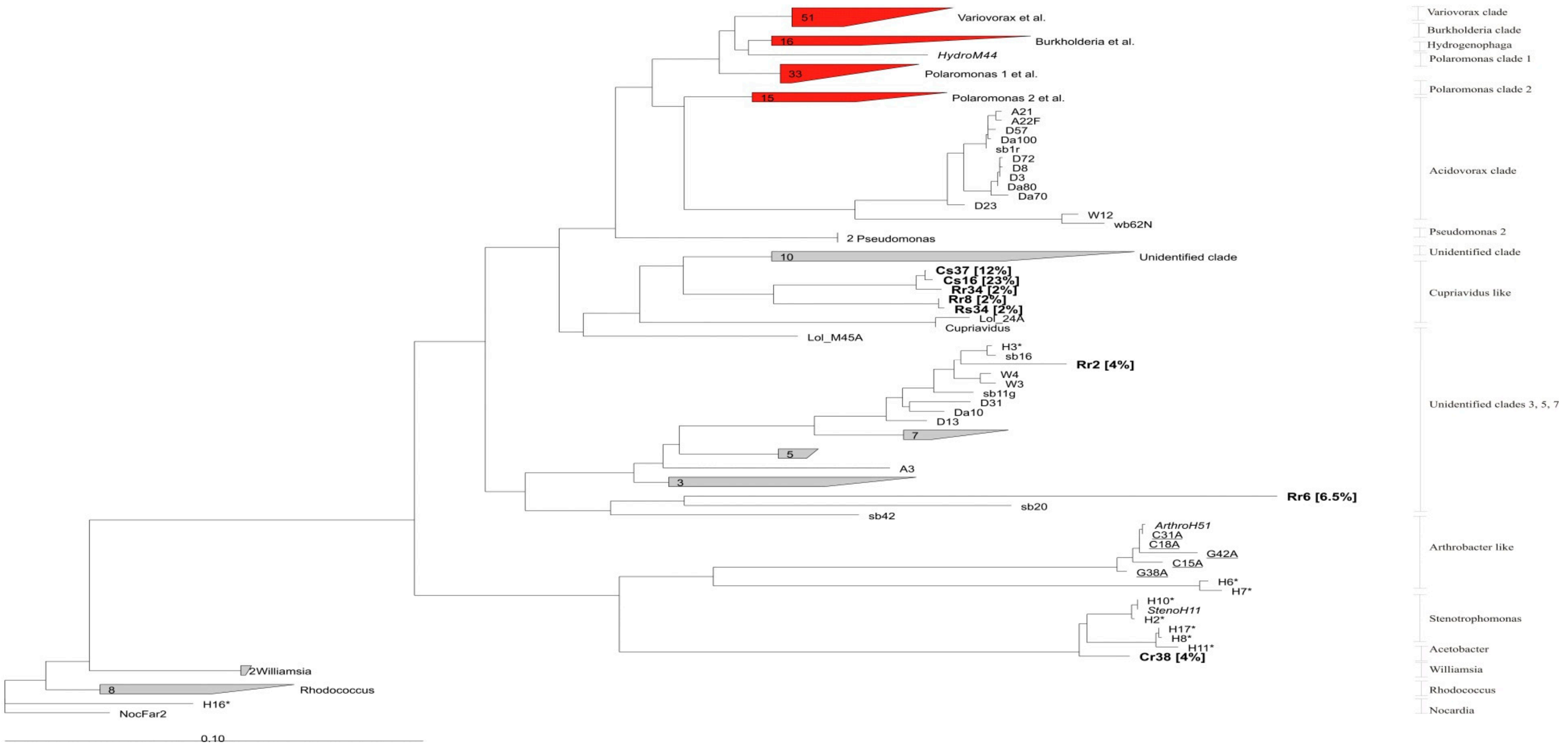

Figure S5 – Randomised axelerated maximum likelihood tree of truncated AsfA sequences of representative desulfonating operational taxonomic units (OTUs) derived from clonal *asfA* DNA sequence analysis from static (CS) and rotating (CR) hyphosphere cores and rhizosphere roots from static = mycorrhizal (RS) and rotating = severed mycorrhizal hyphae (RR) treatments of *A. stolonifera* microcosms. Cultivated (*italics*) and molecular isolates from this study (bold) are highlighted. Molecular isolates from spring barley rhizospheres (sb; [11]), Agrostis grassland rhizospheres (CA; [43]), wheat rhizospheres from Broadbalk (W; [12]), rhizospheres and soils from the Damma glacier forefield (D; DA;[61]) and hyphosphere (H; [15]) were isolated previously. Clades expanded include *Acidovorax, Arthrobacter* and *Stenotrophomonas*. Clades expanded in the preceding figure (Figure S4) are highlighted in red.

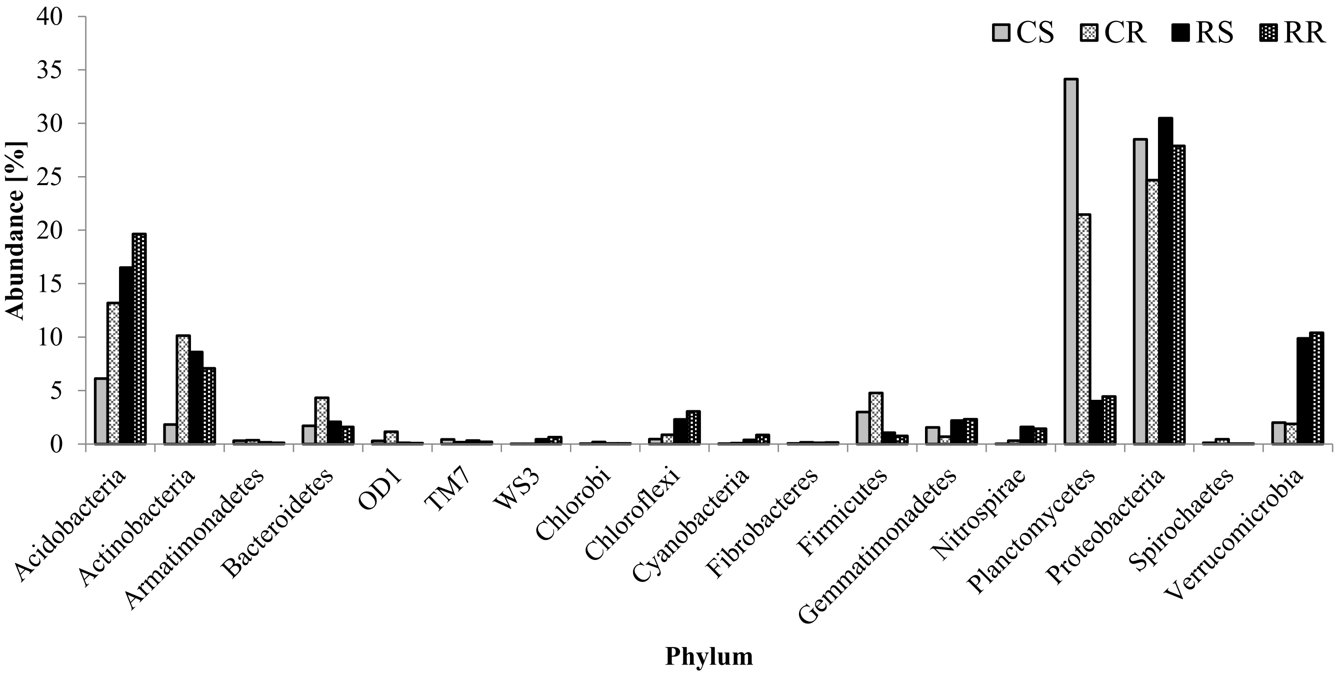

Supplementary Figure S6 – Abundance of sequences allocated to major bacterial phyla (cut-off 0.1%) after taxonomic analysis of 16S rRNA amplicons from hyphosphere cores (C) (grey) and rhizosphere roots (R) (black) of *Agrostis stolonifera* and static (S) = mycorrhizal (solid) and rotating (R) = severed mycorrhizal hyphae (pattern) treatments.

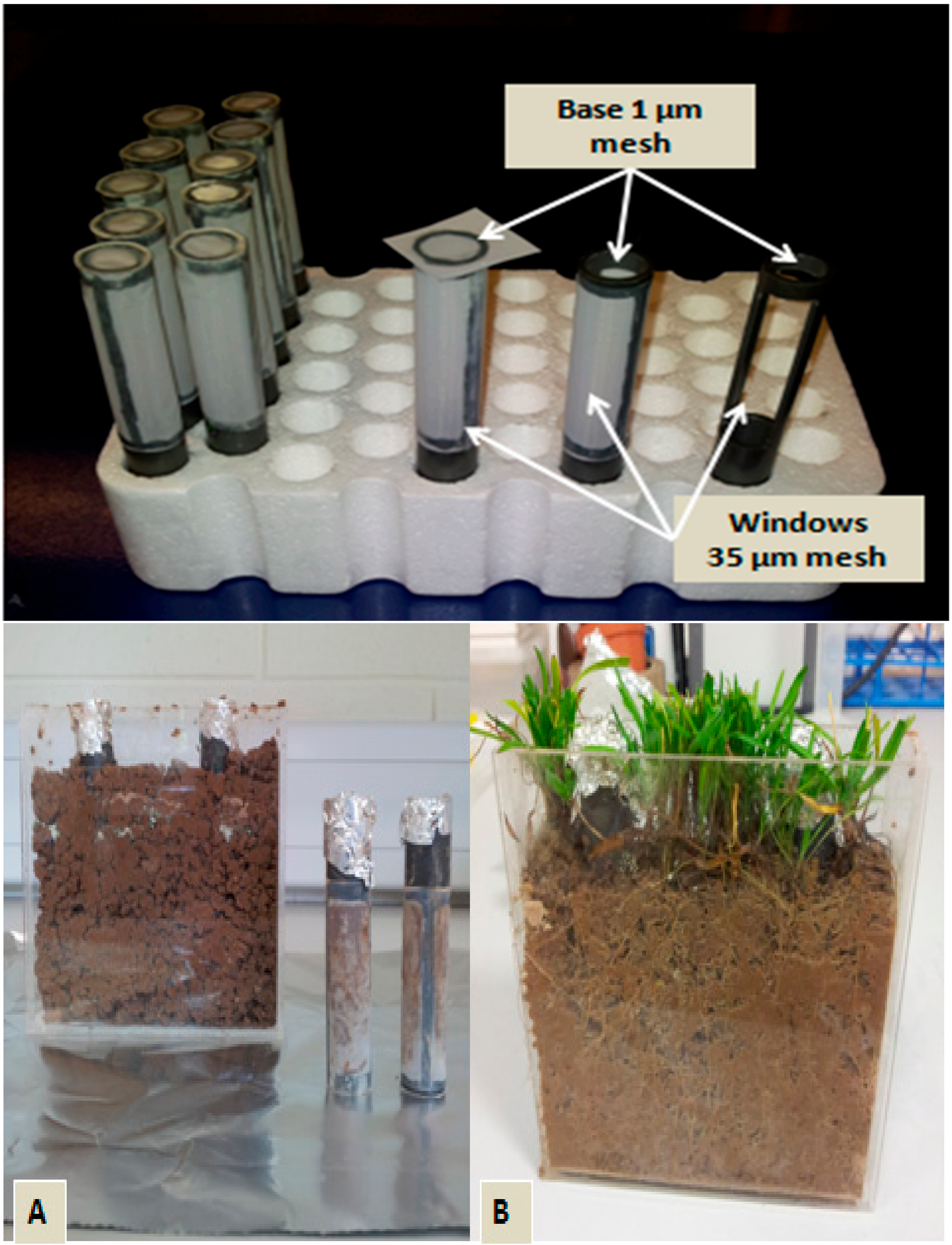

Figure S7: **TOP - Construction of cores used to contain the ^{34}S stable isotope enriched soil. BOTTOM -The soil microcosm systems with the organo-^{34}S enriched cores (A) and the actively growing system (B).**

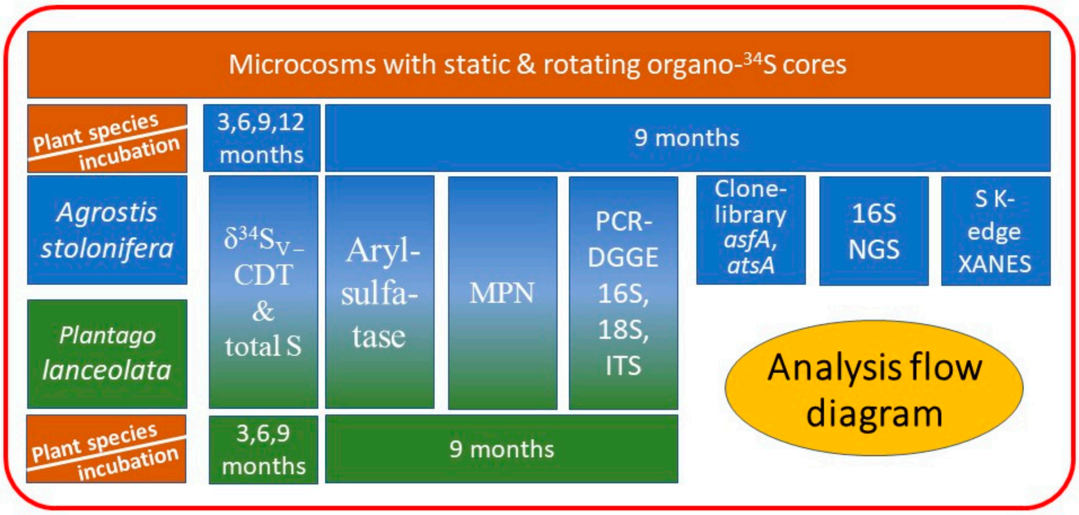

Figure S8: Flow diagram of microcosm sample analysis

Supplementary information - Appendix A

S1 - Determination of ^{34}S uptake

Determination of ^{34}S uptake was achieved using Elemental Analysis - Isotope Ratio Mass Spectrometry (EA-IRMS) undertaken by Iso-Analytical (Chesire, UK). EA-IRMS is a technique used to determine the relative abundance of isotopes in a particular sample. Samples must be introduced to the mass spectrometer as pure gases and this was achieved via combustion. Tin capsules containing reference or sample material plus vanadium pentoxide catalyst were loaded into a furnace (1080 °C). The tin capsules flash combust in the presence of O_2 and the temperature was raised to 1700 °C. Following combustion, the gases were swept in a helium stream over combustion catalysts (tungstic oxide/zirconium oxide) and through a reduction stage of high purity copper wiring to produce SO_2, N_2, CO_2, and H_2O. A Nafion^{TM} membrane was used to remove H_2O and SO_2 was separated from N_2 and CO_2 on a packed GC column (32 °C).

The resultant pure SO_2 entered the ion source of the IRMS where it was ionised and accelerated. Separation of gas species based on mass was achieved in a magnetic field. Simultaneously, the mass-to-charge ration (*m/z*) of the different ion beams was measured on a Faraday cup universal collector array. Analysis was based on monitoring the *m/z* at 48, 49 and 50 of SO^+ ions produced from SO_2 in the ion source to ascertain the relative abundance of ^{32}S (48 *m/z*) to ^{34}S (50 *m/z*). The total S content was calculated from the sum of the ion beams at *m/z* 48, 49 and 50 which represent ^{32}S, ^{33}S and ^{34}S, respectively. Both references and samples were converted to pure SO_2 and analysed using this method.

The reference material used for sulfur isotope analysis was IA-R061 (barium sulfate, $\delta^{34}S_{V\text{-}CDT}$ = +20.33‰). To calibrate and correct for the ^{18}O contribution to the SO^+ ion beam IA-R061, IA-R025 (barium sulfate, $\delta^{34}S_{V\text{-}CDT}$ = +8.53‰) and IA-R026 (silver sulfide, $\delta^{34}S_{V\text{-}CDT}$ = +3.96‰) were used. Delta Units (δ) are expressed in molecules per thousand and are used to denote isotope ratios. For example, if $\delta^{34}S_{V\text{-}CDT}$ = 3.96‰ this means that the sample was analysed against a reference material and found to have 3.96 molecules per thousand more than V-CDT (Vienna-Canyon Diablo Troilite), an iron-sulfide meteorite, which is the accepted zero point for expression of $\delta^{34}S$.

S2 - S K-edge X-ray absorption near edge spectroscopy

Powdered samples were mounted on a sample holder for S K-edge XANES analysis. The monochromator of the beamline was operated in a step by step mode using a Si111 crystal. An ionisation chamber was used to measure the primary flux and a 4 element Si drift detector was used to measure the fluorescence signal. The X-ray energy was calibrated to elemental S at 2472 eV and scans were carried out from 2450 – 2520 eV in steps of 1 eV (2450-2468) and 0.5 eV (2468-2520) (10-20 s per step) in order to identify the different oxidation states of S. Recorded spectra were normalised after base-line subtraction and a linear combination fit was carried out using Athena software (Demeter package 0.9.16) [77].

S3 - Percentage root colonisation

A modified version of the grid line intersect method was used [67] as follows. A representative population of roots (2 root fragments, 10 cm in length were picked from the top, middle, and bottom of the microcosms) and were cut into 1 cm segments. The roots were stained with 20 mL of 10% KOH (w/v) for 12 h. The segments were washed with dH_2O and covered with 20 mL of alkaline H_2O_2 to bleach for 60 min. The bleaching solution was discarded and the roots were rinsed thoroughly with water. Roots were acidified in 20 mL of 0.1 M HCl solution for 12 h to ensure staining of intracellular fungal structures. The HCl solution was discarded and the roots were covered with 20 mL of lactoglycerol trypan blue stain (lactic acid: glycerol: H_2O in a 1:1:1 ratio, with 0.05% (w/v) trypan blue) and incubated at 90 °C for 45 min. The stained roots were then removed and covered in 20 mL of lactoglycerol destain (minus trypan blue) overnight prior to examination. The 1 cm root segments were examined one field of view at a time (x 1000 magnification). The field of view was moved in reference to a graticule inserted into the microscope eyepiece and the point of intersection was determined at the position of the graticule's vertical crosshair entering the root.

Once the point of intersection was noted, the field of view was moved completely through the root and the presence of (1) arbuscules (2) vesicles and (3) hyphae was noted as 'negative' (no fungal structures), 'arbuscules', 'vesicles', or 'hyphae only'. If the crosshair cut an arbuscule or vesicle, the respective category was increased by one and the total number of intersections

was also increased by one. This was also the case for the 'hyphae only' category. In the case of both arbuscules and vesicles being recorded at an intersection, the individual categories were both incremented but the total number of intersections was increased only by one. AC and VC, respectively, were calculated by dividing their respective counts by the total number of intersections examined. HC was calculated as a proportion of the non-negative intersections.

S4 - Minimal Media 2 with Toluenesulfonate or Lignosulfonate

In this project, minimal media 2 with toluenesulfonate (MM2TS) and lignosulfonate (MM2LS) modified by Schmalenberger et al. 2008 were used to cultivate and compare growth rates of sulfonate mobilizing bacteria [12, 6].

10 x Tris stock N

Ingredient	Manufacturer	g/L
Tris	Fisher Scientific	15.138
NH_4Cl	Fisher Scientific	10.7
$MgCl_2$ (1 M)	Fisher Scientific	5 ml

MMtris2 Medium

Ingredient	Manufacturer	mL/L
Water	NA	825
10 x Tris stock N	See above	100
Succinate (1 M)	Fisher Scientific	5
Glycerol (50 %) (v/v)	Fisher Scientific	980 µL
Sodium /Potassium Chloride (1 M)	Fisher Scientific	20
Potassium Phosphate (1 M)	Fisher Scientific	10
200 x Trace elements	See below	5
Toluene Sulfonate (100 mM, pH 7) **MM2TS**	Merck	2.5
Lignosulfonate (100mM, pH 7) **MM2LS**	Aldrich	5.96
$^{\alpha}$Fructose (0.5 M)	VWR	5.6

$^{\alpha}$added after separate autoclaving

Trace Elements Stock

The trace element solution used was originally designed for cultivation of phototrophic sulfur bacteria [78].

Trace Elements Stock

Ingredient	mg/L
$Na_2EDTA.2H_2O$	500
$FeCl_2.4H_2O$	143
$ZnCl_2$	4.7
$MnCl_2.4H_2O$	3
H_3BO_3	30
$CoCl_2.2H_2O$	20
$CuCl_22H_2O$	1
$NiCl_2.6H_2O$	2
$Na_2MoO_{4.}2H_2O$	3
$CaCl_2.2H_2O$	100

The MM2TS, MM2LS solution was autoclaved for 15 min at 121° C for sterilisation. Subsequently, 5.6 ml/L of 0.5 M sterile fructose (pH 7.2) was added to the mixture. The fructose is added after autoclaving to prevent decomposition of the compound. Additionally, for solid media, 4.8 g/L of molecular grade agarose (Eurobio, France) was added prior to autoclaving.

S5 – PCR-DGGE

Bacterial 16S rRNA gene amplification was carried out with this DNA using the primer pair GC-341F/518R (supplementary Table S1) targeting the V3 region for DGGE {Muyzer, 1993 #36}. The final concentration per 25 µL reaction was 1 X buffer (2 mM $MgCl_2$), 0.2 mM dNTP mix, 0.4 µmol of each primer, and 0.5 U of DreamTaq polymerase (Fermentas, Waltman, MA). A touchdown PCR protocol was used with the following cycling conditions: initial denaturation of 94 °C for 5 min, 20 cycles of 94 °C denaturation (45 s), 65-55 °C touchdown (45 s), 72 °C extension (45 s), plus 18 further cycles with an annealing temperature at 55 °C. Final extension was carried out at 72 °C for 5 min. DGGE was carried out on 200 x 200 x 1

mm gels in a TV400 DGGE apparatus (Scie-Plas, Cambridge, UK). Gels of 10% (w/v) acrylamide/bisacrylamide were prepared and run using a linear 30-60% gradient in 1 X TAE buffer (60 °C) for 16.5 h at 63 V [14]. After completion, gels were stained with SYBR Gold (1:10,000 diluted) (Invitrogen, Carlsbad, CA) for 30 min and the image captured on a Syngene G:Box (Cambridge, UK).

AM fingerprinting was achieved using the AM specific primer AM1 [80] alongside the universal eukaryotic primer NS31 [81] targeting 18S rRNA. The PCR were undertaken in 25 μL reactions with 1 X buffer (2 mM $MgCl_2$), 1 M betaine, 0.2 mM dNTP mix, 0.4 μmol of each primer and 0.5 U of DreamTaq polymerase (Fisher Scientific, Waltham, MA). The PCR was carried out under the following conditions: initial denaturation of 94 °C for 5 min, 30 cycles of 94 °C denaturation (30 s), 58 °C annealing (60 s), and 72 °C extension (90 s). Final extension was carried out at 72 °C for 5 min. A tenfold dilution of the PCR product was undertaken and used as template for a nested PCR using the primer set Glo1 [82] and NS31-GC [83]. The nested PCR was carried out under the following conditions: initial denaturation of 94 °C for 5 min, 20 cycles of 94 °C denaturation (45 s), 58-48 °C touchdown (45 s), 72 °C extension (45 s), plus 15 further cycles with an annealing temperature at 48 °C. Final extension was carried out at 72 °C for 5 min. DGGE was run as before with a gradient of 35-55%.

Fungal DNA fingerprinting was undertaken using the fungal specific primer ITS-1F [84] and ITS-4 [85]. This product was tenfold diluted and used as template in a nested PCR using an ITS-1FGC primer with a 40 base GC clamp to the 5' end of the primer [86] and ITS-2 reverse primer [85]. Both PCRs were undertaken in 25 μL reactions with 1 X buffer (2 mM $MgCl_2$), 1 M betaine, 0.2 mM dNTP mix, 0.4 μmol of each primer and 0.5 U of DreamTaq polymerase (Fisher Scientific, Waltham, MA). For both PCR reactions, amplification was performed with an initial denaturation of 94 °C for 5 min, 40 cycles with the first 20 cycles at 95 °C denaturation (45 s), 60 °C annealing (45 s), and 72 °C extension (45 s). Cycles 21–40 used the same parameters with annealing temperature of 50 °C [84]. Final extension was carried out at 72 °C for 5 min. DGGE was run as before with a gradient of 35-65%.

S6 - Arylsulfatase activity

Acetate buffer (0.5 M, pH 5.8) was prepared by dissolving 64 g of sodium acetate trihydrate in 200 mL dH_2O, adding 1.70 mL glacial acetic acid (99%) and diluting this to 1 L. *p*-Nitrophenol solution (500 μg/L) was prepared in acetate buffer. For analysis, 1 g of soil was placed in 15

mL centrifuge tubes with 4 mL of acetate buffer, 0.25 mL of toluene and 1 mL of 20 mM *p*-nitrophenyl sulfate. The contents were vigorously mixed and incubated at 37 °C for 1 h (rotated 360 degrees at 10 minute intervals). After the incubation, 2 mL of 1 M NaOH and 1 mL of 0.5 M $CaCl_2$ were added to stop the reaction. The centrifuge tubes were subjected to centrifugation (10 min, 4500 rpm). The absorbance of the supernatant was recorded at 400 nm in a UV MINI spectrophotometer (Shimadzu, Japan). The *p*-Nitrophenol content of the treatments was determined in reference to a calibration curve with *p*-Nitrophenol [71]. This curve was obtained by preparing 100 mL of *p*-Nitrophenol solution (500 µg/L), of this 0, 1, 2, 3, 4, and 5 mL aliquots were prepared and made up to 5 mL in H_2O to generate the 0, 10, 20, 30, 40, and 50 µg *p*-Nitrophenol standards. At this point, the standards were treated as described above for the post incubated soil samples and the absorbance of the supernatant was recorded at 400 nm in a UV MINI spectrophotometer (Shimadzu, Japan).

Table S1

Primers used in this study

ID	Sequence	Target	Reference
27F	5'-AGAGTTTGATCMTGGCTCAG-3'	16S Rrna	[87]
1492R	5'-GGTTACCTTGTTACGACTT-3'	16S Rrna	[87]
AsfBtoA	5'-ASCTCGCACATGAAGCAGG-3'	*asfA* gene	[11]
asfAF1all	5'-YTSTCVGGCATGGAGTTYT-3'	*asfA* gene	[15]
AtsA-F1	5'-TIGCIGAYGAYITSGGITWYTCTGA-3'	*atsA* gene	[9]
AtsA-R1	5'-TCSGSICCRTTGTCSGACATGAA-3'	*atsA* gene	[9]
341F-GC	5'CGCCCGCCGCGCGCGGCGGGCGGGGCGGGGGCAC GGGGGGCCTCGGGACCGAGCAG-3'	16S rRNA	[79]
518R	5'-ATTACCGCGGCTGCTGG-3'	16S rRNA	[79]
Glo1	5'-GCCTGCTTTAAACACTCTA-3'	18S rRNA	[82]
NS31-GC	5'CGCCCGGGGCGCGCCCCGGGCGGGGCGGGGGCAC GGGGGTTGGAGGGAGGGCAAGTCTGGTGCC-3'	18S rRNA	[83]
ITS1F	5'-CTTGGTCTTTAGAGGAAGTAA-3'	ITS fungal	[84]
ITS4	5'-TCCTCCGCTTATTGATATGC-3'	ITS fungal	[85]
ITS1F-GC	5'CGCCCGCCGCGCGCGGCGGGCGGGGCGGGGGCGC GGGGGCCTTGGTCTTTAGAGGAAGTAA-3'	ITS fungal	[86]
ITS2	5'-GCTGCGTTCTTCATCGATGC-3'	ITS fungal	[85]
AM1	5'-GTTTCCCGTAAGGCGCCGAA-3'	18S AM fungal	[80]
NS31	5'-TTGGAGGGCAAGTCTGGTGCC-3'	18S rRNA	[81]
16SF	5'TCGTCGGCAGCGTCAGATGTGTATAAGAGACAGCC TACGGGNGGCWGCAG 3'	16S (V3-V4 region)	Illumina Metagenomic Sequencing Library Preparation Guide
16SR	5'CTCTCGTGGGCTCGGAGATGTGTATAAGAGACGAG ACTACHVGGGTATCTAATCC -3'	16S (V3-V4 region)	Illumina Metagenomic Sequencing Library Preparation Guide

References, see main manuscript
